# Evaluating risk factors for surgical site infections following open reduction and internal fixation surgery for ankle fractures: a systematic review and meta-analysis

**DOI:** 10.3389/fpubh.2025.1558994

**Published:** 2025-07-17

**Authors:** Feifan Luan, Cong Zheng, Yuzhong Zheng, Jiayi Chen, Yurui Wu, Chenxiao Zheng

**Affiliations:** ^1^The Tenth Clinical Medical College of Guangzhou University of Traditional Chinese Medicine, Zhongshan Hospital of Traditional Chinese Medicine Affiliated to Guangzhou University of Traditional Chinese Medicine, Zhongshan, Guangdong Province, China; ^2^Key Laboratory of Biological Targeting Diagnosis, Therapy and Rehabilitation of Guangdong Higher Education Institutes, The Fifth Affiliated Hospital, Guangzhou Medical University, Zhongshan, Guangdong Province, China

**Keywords:** ankle fractures, surgical site infections, open reduction and internal fixation, risk factors, meta-analysis

## Abstract

**Background:**

Ankle fractures are common injuries requiring surgical intervention, specifically open reduction and internal fixation (ORIF), which carries a risk of surgical site infections (SSIs). Identifying and understanding the risk factors associated with SSIs in these patients is crucial for improving surgical outcomes and patient care.

**Methods:**

This systematic review and meta-analysis followed the Preferred Reporting Items for Systematic Reviews and Meta-Analyses (PRISMA) guidelines. We searched PubMed, Embase, Web of Science, and the Cochrane Library on September 19, 2023, without restrictions on publication period or language. The inclusion criteria comprised observational studies and randomized controlled trials that investigated risk factors for SSIs following ORIF for ankle fractures. Exclusion criteria included non-empirical studies, studies without specific outcomes on SSIs, non-ORIF surgeries, and studies with incomplete data. The quality of included studies was assessed using the Newcastle-Ottawa Scale (NOS), and statistical analyses were performed using Stata 17.

**Results:**

Out of 1,255 initially identified articles, eight studies met the inclusion criteria after deduplication, screening, and full-text review. These studies highlighted several risk factors for SSIs, including diabetes, open fractures, high-energy injuries, and smoking, with diabetes and open fractures significantly increasing the risk. Antibiotic prophylaxis emerged as a protective factor. The quality assessment revealed a high standard of research quality among the included studies, and sensitivity analysis confirmed the robustness of the findings.

**Conclusion:**

This meta-analysis underscores the importance of recognizing diabetes, open fractures, high-energy injuries, smoking, and a BMI exceeding 30 as significant risk factors for SSIs following ORIF for ankle fractures. The administration of antibiotic prophylaxis serves as a protective measure. Healthcare providers should incorporate strategies to mitigate these risks, enhancing postoperative care and reducing the incidence of surgical site infections.

## 1 Introduction

Ankle fractures are frequently encountered in emergency departments globally and require surgical intervention, particularly open reduction and internal fixation (ORIF), to achieve optimal anatomical realignment and functional recovery (1, 2). Although this surgical technique has played a crucial role in improving outcomes for patients with fractures of this nature, it is not without its share of consequences, with surgical site infections(SSIs) being particularly noteworthy as a serious issue. SSIs not only extend the duration of hospital stays and escalate healthcare expenses, but also present significant difficulties in patient care, potentially resulting in delayed wound healing, osteomyelitis, and, in severe instances, the requirement for supplementary surgical procedures (3, 4).

The reported occurrence of SSIs after ORIF for ankle fractures exhibits considerable diversity, highlighting the intricate interaction of various factors that requires a further examination. The susceptibility to infections can be influenced by various patient-related characteristics, including age, gender, underlying health disorders such as diabetes or peripheral vascular disease, as well as lifestyle factors such as smoking and nutritional status (5). Perioperative parameters, which include the surgical environment, duration of the surgery, type of anesthesia, and adherence to aseptic methods, are crucial in determining the risk of infection. The presence of diverse research methodologies, including prospective cohort studies and retrospective analyses, as well as the inclusion of different patient cohorts ranging from pediatric to geriatric populations, and the utilization of varying definitions of surgical site infections, ranging from superficial to deep infections, pose challenges in making direct comparisons between studies (6, 7). This landscape requires a methodical and comprehensive approach to combine different findings, providing a complete understanding of the factors that contribute to SSIs in this surgical setting. This will help informing clinical practices and guidelines that are based on evidence, with the goal of reducing infection rates and improving patient outcomes.

The main aim of this systematic review and meta-analysis is to conduct a thorough assessment of the current literature in order to identify and measure the risk factors linked to the occurrence of SSI in patients who are undergoing ORIF for ankle fractures.

## 2 Methods

### 2.1 Search strategy

In conducting our systematic review, we adhered to the Preferred Reporting Items for Systematic Reviews and Meta-Analyses (PRISMA) guidelines to ensure a structured and transparent approach (8). The literature search was conducted on September 19, 2023, across four major electronic databases: PubMed, Embase, Web of Science, and the Cochrane Library, without imposing any restrictions on the publication period. We employed a search strategy, utilizing key terms such as ‘factor,’ ‘predictor,’ ‘risk,’ ‘infection,’ and ‘ankle fractures.’ These terms were chosen to align with the PICO (Patient, Intervention, Comparison, and Outcome) framework, aiming to capture a range of studies pertinent to our meta-analysis. The search was inclusive, with no language restrictions, to maximize the scope of the investigation. The detailed search strategies for each database are provided in Supplementary Table 1. Additionally, we conducted a manual screening of the reference lists of all identified articles to uncover any further studies that could contribute to our analysis, ensuring no potential sources of relevant data were overlooked.

### 2.2 Inclusion and exclusion criteria for meta-analysis

Inclusion criteria:

1) Study design: we included observational studies (cohort, case–control, and cross-sectional studies) and randomized controlled trials that investigated risk factors for surgical site infections following open reduction and internal fixation (ORIF) for ankle fractures.
2) Population: studies involving patients of any age group who underwent ORIF for ankle fractures were considered. Ankle fractures were defined as injuries involving the distal tibia and/or fibula around the ankle joint.
3) Outcome measures: only studies that explicitly reported on surgical site infections as an outcome, with clear diagnostic criteria for infections, were included.
4) Risk factors: studies needed to examine one or more risk factors associated with the development of surgical site infections to be included.

Exclusion criteria:

1) Non-empirical studies: reviews, editorials, commentaries, and case reports were excluded to ensure the inclusion of only empirical data.
2) Studies without specific outcomes: studies that did not specifically report on surgical site infections as a distinct outcome were excluded.
3) Non-ORIF surgeries: studies focusing on patients treated with methods other than open reduction and internal fixation for ankle fractures were not included.
4) Incomplete data: studies lacking sufficient data on risk factors or outcomes, and those without available full texts, were excluded to ensure the reliability and completeness of the data analyzed.
5) Studies involving multiple types of lower extremity fractures (e.g., femur, tibia, and ankle) were excluded, even if they included subgroup analyses for ankle fractures, in order to reduce clinical heterogeneity and maintain specificity of the pooled estimates.

### 2.3 Data extraction process for meta-analysis

In our meta-analysis, the literature screening and data extraction process was conducted by two independent evaluators to ensure accuracy and objectivity. Each evaluator performed the extraction autonomously, with their findings then cross-verified to confirm consistency. Discrepancies encountered were addressed through discussion between the reviewers to reach a consensus. When an agreement could not be achieved, a third reviewer was consulted for an impartial resolution. The extracted data included the author(s) of each study, the publication year, the total number of cases assessed, the country of the study, the age range of the participants, the type of study (e.g., cohort, case–control, randomized control trial), and any significant factors identified as influencing the risk of surgical site infections in patients undergoing open reduction and internal fixation for ankle fractures. When specific data of interest was missing from the published reports, efforts were made to obtain this information directly from the original investigators, which involved reaching out to the authors via email to request any relevant unpublished data, ensuring a comprehensive dataset for analysis.

### 2.4 Quality assessment of included studies

To ensure the reliability and validity of our meta-analysis, the quality of each included study was assessed by two reviewers using the Newcastle-Ottawa Scale (NOS), designed for the appraisal of non-randomized studies (9). The NOS framework includes nine items across three domains: selection of study groups, comparability of groups, and ascertainment of the exposure or outcome for case–control or cohort studies, respectively. These domains help identify potential biases within the included studies. During the assessment, each criterion within the NOS was assigned an asterisk, with each asterisk contributing one point toward the study’s overall quality score. This scoring system allowed a structured evaluation, resulting in a total quality score for each study ranging from 0 to 9 points. Studies achieving a score from 0 to 3 points were categorized as low quality, indicating significant methodological limitations. Studies scoring between 4 and 6 points were classified as moderate quality, suggesting some methodological concerns. In contrast, studies scoring from 7 to 9 points were recognized as high quality, indicative of robust methodological approaches and a lower risk of bias.

### 2.5 Statistical analyses

In the meta-analysis, Stata 17 was utilized for statistical evaluation, starting with heterogeneity analysis among studies via chi-square tests and I^2^ statistics. Heterogeneity was considered negligible when I^2^ was under 50% with a *p*-value ≥ 0.10, prompting the use of a fixed-effect model for effect size calculation. Conversely, significant heterogeneity, indicated by an I^2^ ≥ 50% or *p*-value < 0.10, necessitated a random-effects model to account for variability beyond chance (10). Sensitivity analysis, through the sequential removal and reevaluation of studies, was critical for assessing the robustness of the results and identifying outlier studies. Publication bias was evaluated using Egger’s linear regression test. The meta-analysis adhered to a two-sided statistical testing approach, with a significance threshold set at *p* < 0.05, ensuring the findings’ reliability and validity.

## 3 Results

### 3.1 Search results and study selection

**Figure 1:**
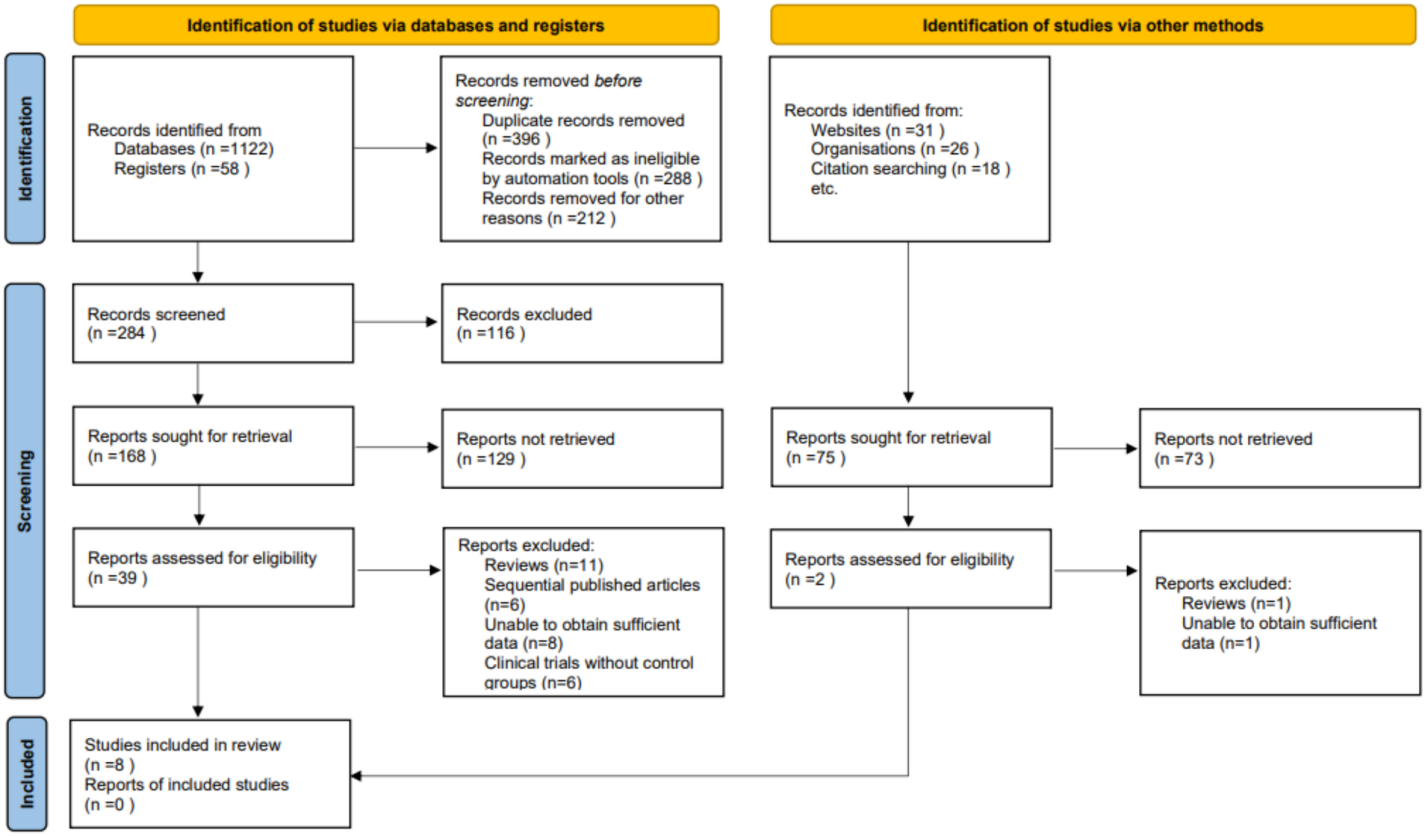
Study selection flowchart for meta-analysis inclusion.

During the initial phase of our systematic review and meta-analysis, a thorough search across various databases yielded 1,255 articles potentially relevant to our topic. Subsequent deduplication efforts resulted in the removal of redundant entries, leaving a streamlined collection for further assessment. The screening of titles and abstracts, guided by clearly defined inclusion and exclusion criteria, led to the selection of 41 articles for detailed examination. These criteria spanned study design, participant demographics, measured outcomes, and research quality. Independent review of the full texts of these articles by multiple investigators further narrowed the field, excluding 33 articles due to reasons such as being review articles (12), publications in sequence (6), lack of sufficient data (9), and absence of control groups in clinical trials (6). Ultimately, eight studies were found to align with our stringent selection criteria and were incorporated into the final meta-analysis (3, 11–17) (Figure 1).

### 3.2 Study characteristics

The meta-analysis incorporated studies spanning from 1999 to 2017, reflecting a diverse array of research focused on surgical site infections following orthopedic procedures. These studies were predominantly retrospective cohort studies, with a single case–control study and one prospective cohort study, indicating a reliance on observational data for insights into this clinical issue. The research emanated from a global context, with studies conducted in China, Norway, Denmark, Singapore, the United Kingdom, Sweden, and Japan, highlighting the widespread interest and relevance of postoperative infection risks across different healthcare systems. The participant age range varied widely among the studies, from as young as 9 to as old as 95 years, underscoring the broad demographic impact of orthopedic surgical interventions and their associated complications. Control and case groups across these studies were sizeable, with the largest control group comprising 1,444 participants and the smallest having 37, reflecting significant variability in study scale. Case groups also varied, from 8 to 146 individuals, indicating differences in the incidence and reporting of postoperative infections across different settings and populations. Overall, the included studies offer a comprehensive overview of the factors influencing surgical site infections in orthopedic surgery patients, providing valuable insights derived from a broad spectrum of patient populations and healthcare environments (Table 1).

**Table 1:** Basic characteristics of included studies.

Author	Year	Control group	Case group	Age range	Country	Study type	Risk factors / outcome measurement
Sun R (3)	2017	1,444	66	18–86	China	Retrospective cohort	Diabetes, High-energy mechanism, Incision cleanness 2–4, Open injury, Preoperative NEUT >75%, CHD, Surgeon level
Sun Y (16)	2017	1,201	46	18–81	China	Retrospective cohort	Diabetes, BMI, Surgical duration >130 min, Surgeon level, Delayed surgery, Preoperative TP < 60
Naumann (14, 24)	2016	538	29	NA	Norway	Retrospective cohort	Age, Sex, ASA class, Smoking, Diabetes, Fracture classification, Preoperative antibiotics
Olsen (15)	2016	897	146	9–95	Denmark	Retrospective cohort	Obesity, Alcohol overuse, Smoking, Gender, Diabetes, ASA score, Fracture type, Age
Tan (17)	2015	37	8	Mean: 62	Singapore	Retrospective cohort	Age, Sex, Operating time, Adequacy of fixation, Type of diabetic control, Complicated diabetes
Korim (12)	2014	681	29	Mean: 51	United Kingdom	Case–control	Diabetes, Nursing home residence, Weber C, Open fracture, Smoking, Alcohol, Dislocation >50%, External fixator, etc.
Nasell (13)	2011	795	111	NA	Sweden	Prospective cohort	Smoking, Fracture type, Timing of surgery
Blotter (11)	1999	58	9	NA	Japan	Retrospective cohort	Diabetes

NA, Not available.

### 3.3 Quality assessment results of included studies

The quality assessment of the cohort studies included in our meta-analysis, based on the NOS, revealed a generally high standard of research quality among the selected studies. The evaluation covered three critical domains: Selection, Comparability, and Outcome, with a maximum attainable score of 9 points across these categories. Most studies demonstrated a robust methodology, particularly in the Selection and Outcome categories, where the majority received full marks. This indicates strong representativeness of the exposed cohorts, clear ascertainment of exposure, and thorough assessment of outcomes, alongside ensuring that the outcomes of interest were not present at the study’s start. Comparability, based on the design or analysis, was another area where several studies excelled, with many receiving the maximum score available in this category. This reflects the rigorous effort to control for confounding factors and ensure that comparisons between cohorts were valid and reliable. Overall, scores ranged from 7 to 9, with several studies achieving the maximum score, underscoring the high quality of the included research (Table 2).

**Table 2:** The quality assessment according to Newcastle-Ottawa scale of each cohort study.

Study	Selection			Comparability		Outcome			Total score
	Representativeness of the exposed cohort	Selection of the non-exposed cohort	Ascertainment of exposure	Demonstration that outcome of interest was not present at start of study	Comparability of cohorts on the basis of the design or analysis	Assessment of outcome	Was follow-up long enough	Adequacy of follow up of cohorts	
Sun R (3)	★	★	★	★	★★	★	★	★	9
Sun Y (16)	★	★		★	★	★	★	★	7
Naumann (14, 24)	★		★	★	★	★	★	★	7
Olsen (15)	★	★	★	★	★	★	★	★	8
Tan (17)	★	★	★	★	★★	★	★	★	9
Korim (12)		★	★	★	★★	★	★	★	8
Nasell (13)	★	★	★	★	★★	★		★	8
Blotter (11)	★	★	★	★	★★	★	★	★	9

★Each individual asterisk (‘★’) signifies one point.

### 3.4 Impact of diabetes on surgical site infections

**Figure 2:**
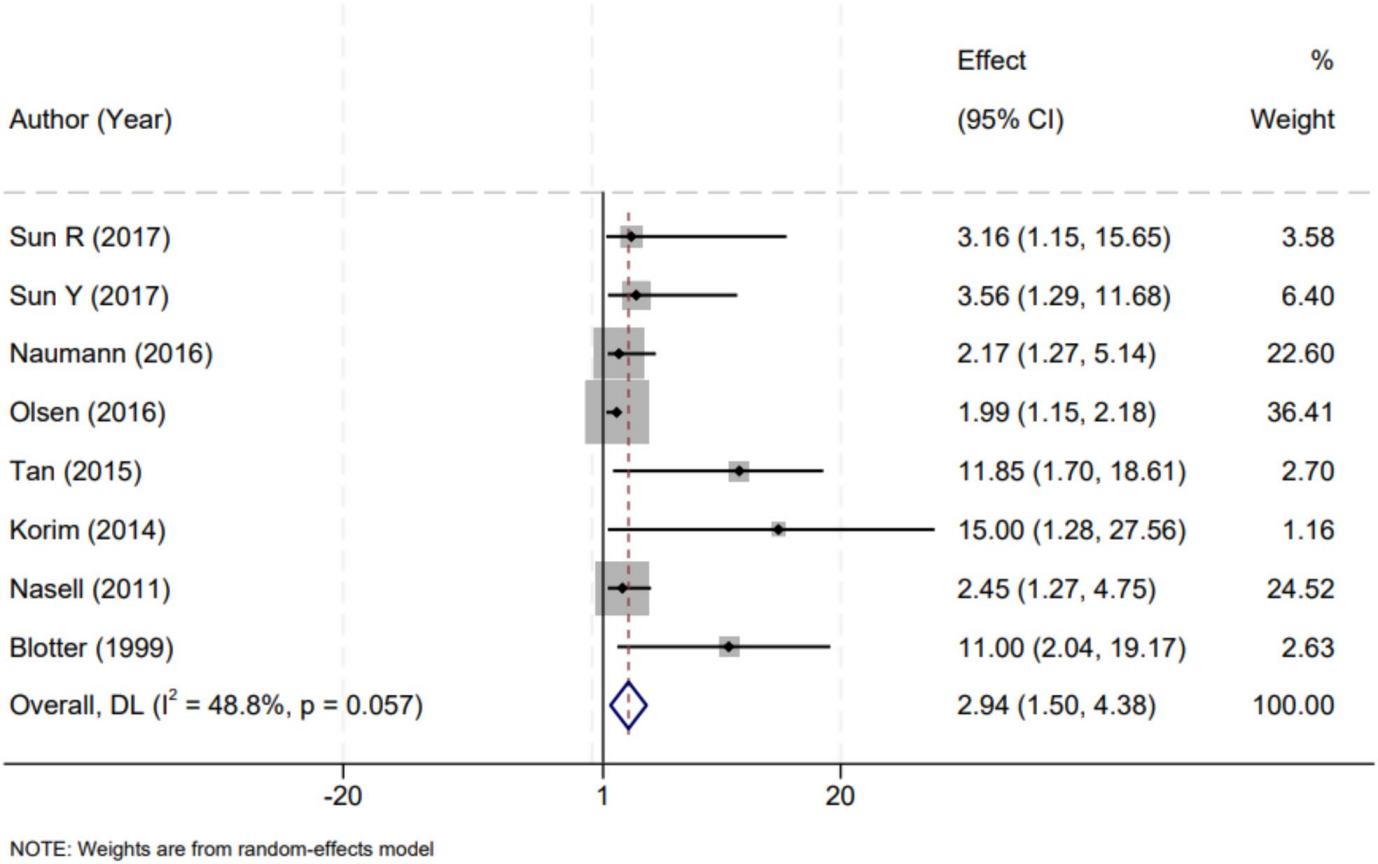
Forest plot demonstrating the association between diabetes and the risk of surgical site infections following open reduction and internal fixation for ankle fractures. The effect measure is presented as odds ratio (OR) with corresponding 95% confidence intervals (CI). Each square indicates the point estimate of an individual study, with its size reflecting the study’s weight in the meta-analysis. The horizontal lines represent 95% CIs. The diamond represents the overall pooled effect. The vertical dashed line at OR = 1 denotes the line of no effect. DL refers to the DerSimonian–Laird method for random-effects pooling; IV refers to the inverse-variance method used for fixed-effects models (when applicable). Statistical heterogeneity was assessed using Cochran’s Q-test and quantified by the I^2^ statistic.

In the meta-analysis, eight studies were identified that specifically reported on the impact of diabetes on the incidence of SSIs. The analysis of these studies revealed considerable heterogeneity (I^2^ = 48.8%, *p* = 0.057), necessitating the use of a random-effects model to account for the variability among study results. The pooled effect size, expressed as an odds ratio (OR), was calculated to be 2.94 with a 95% confidence interval (CI) ranging from 1.50 to 4.38. This statistically significant result (*p* < 0.01) indicates that diabetes is associated with an increased risk of developing SSIs following surgical procedures. The OR of 2.94 suggests that patients with diabetes are almost three times more likely to develop a postoperative surgical site infection compared to non-diabetic patients. This finding underscores the importance of rigorous perioperative care and monitoring for patients with diabetes undergoing surgery, particularly given the substantial role diabetes may play in postoperative complications (Figure 2).

### 3.5 Influence of open fractures on surgical site infections

**Figure 3:**
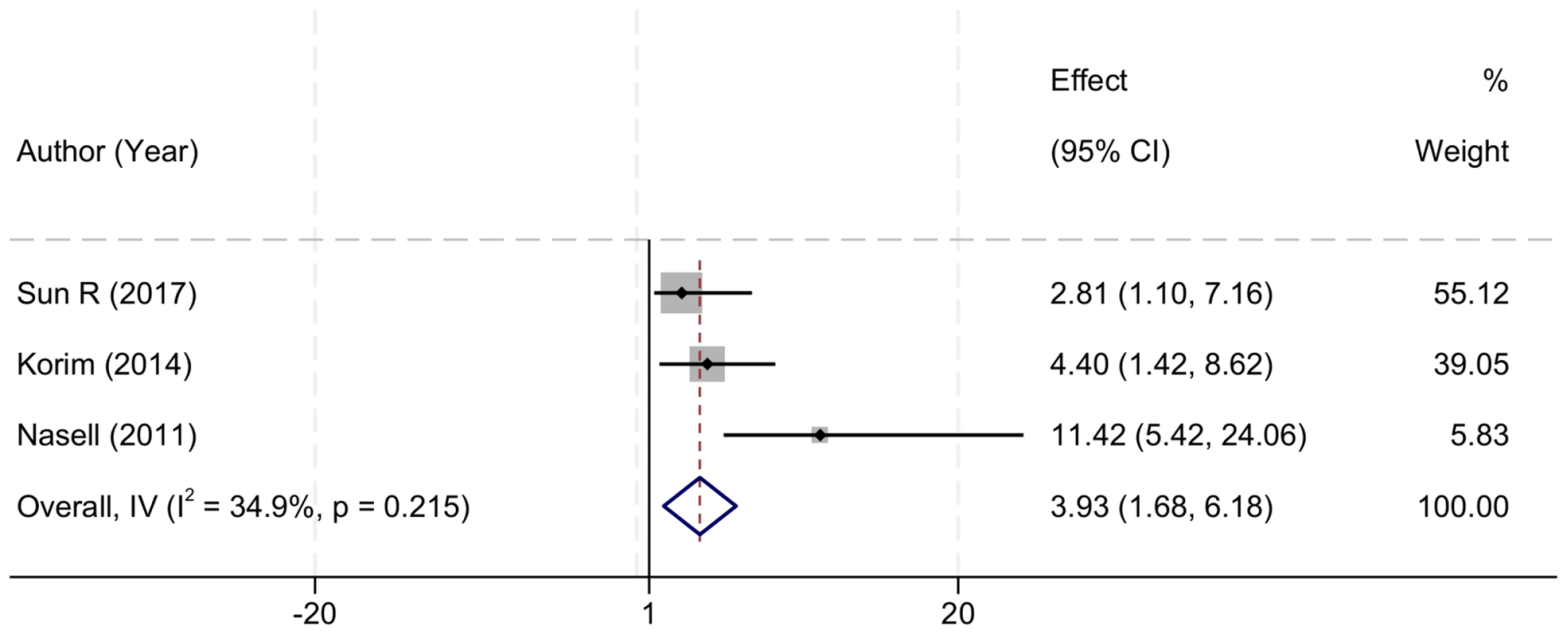
Forest plot demonstrating the association between open fractures and the risk of surgical site infections following open reduction and internal fixation for ankle fractures. The effect measure is presented as odds ratio (OR) with corresponding 95% confidence intervals (CI). Each square represents the point estimate of an individual study, with the size proportional to its statistical weight. The horizontal lines indicate the 95% CI for each study. The diamond represents the pooled effect estimate. The vertical dashed line at OR = 1 denotes the line of no effect. IV refers to the inverse-variance method used for fixed-effects pooling. Statistical heterogeneity was assessed using Cochran’s Q-test and quantified with the I^2^ statistic.

Our meta-analysis scrutinized the relationship between open fractures and the development of SSIs, synthesizing data from three studies that explored this association. The analysis determined a low to moderate level of heterogeneity among the selected studies (I^2^ = 34.9%, *p* = 0.215), which justified the application of a fixed-effect model for the estimation of the combined effects. The aggregated data yielded an OR of 3.93 with a 95% CI of 1.68 to 6.18, indicating a statistically significant increase in the risk of SSIs associated with open fractures (*p* < 0.01). This effect size implies that patients with open fractures are nearly four times more likely to experience a postoperative surgical site infection compared to those with closed fractures. These findings highlight the critical need for enhanced infection control protocols and vigilant postoperative management for patients presenting with open fractures (Figure 3).

### 3.6 Various risk factors affecting surgical site infections

In our meta-analysis, various risk factors were analyzed for their influence on postoperative complications. External fixator usage, with a high odds ratio, was identified as a notable risk, suggesting a substantial increase in the likelihood of complications, while high-energy injury mechanisms significantly raised postoperative risks, as evidenced by their elevated odds ratios. The cleanliness of the incision site was another critical determinant, with improved cleanliness markedly reducing the odds of adverse outcomes. Patient-related factors such as higher ASA scores, alcohol consumption, and male gender were associated with a greater risk of complications, indicating the importance of individual health status in surgical recovery. Chronic heart disease and conditions like subluxation/dislocation also affected postoperative outcomes, reinforcing the need for careful management of comorbidities. Longer surgical times and smoking were correlated with increased risks, whereas factors like higher BMI, hypertension, and female gender had a less pronounced but still noteworthy impact on complication rates. Interestingly, drainage usage and surgeon experience level did not significantly alter the risk landscape according to our findings. Delaying surgery for more than 24 h and having a history of previous surgeries at any site showed a trend toward lower odds of complications, though these findings were not statistically significant. The use of antibiotic prophylaxis emerged as a protective factor, significantly lowering the risk of postoperative issues (Table 3). Overall, these risk factors paint a complex picture of the postoperative environment, where both patient characteristics and surgical decisions contribute to the outcome. Understanding these factors can lead to improved strategies for managing the risks associated with surgery.

**Table 3:** Summary of meta-analysis findings.

Risk factor	Number of studies	Pooled odds ratio	LL 95% CI	UL 95% CI	*p*-value	Q-test (P)	I^2^ (%)	Model
External fixator usage	2	11.249	0.079	1615.277	0.318	0.002	86.400	Random-effects model
High-energy injury mechanism	2	4.074	1.455	11.407	0.006	0.008	82.464	Random-effects model
Incision cleanliness	2	3.252	2.110	5.009	<0.001	0.227	27.648	Fixed-effects model
ASA ≥ 3	2	3.198	1.780	5.745	<0.001	0.874	0.000	Fixed-effects model
Alcohol consumption	3	2.240	0.982	5.106	0.042	0.095	54.528	Random-effects model
Male gender	2	2.145	0.497	9.250	0.270	0.005	83.904	Random-effects model
Chronic heart disease	2	2.141	0.964	4.756	0.047	0.940	0.000	Fixed-effects model
Subluxation/dislocation	2	1.920	1.189	3.100	0.005	0.960	0.000	Fixed-effects model
Surgical time > 90 minutes	2	1.789	0.698	4.588	0.187	0.129	53.280	Random-effects model
Smoker	5	1.560	0.909	2.678	0.075	0.004	70.752	Random-effects model
BMI > 30	2	1.546	1.021	2.340	0.023	0.178	41.280	Fixed-effects model
Hypertension	2	1.360	0.810	2.283	0.180	0.444	0.000	Fixed-effects model
Female gender	3	1.140	0.894	1.454	0.160	0.333	5.376	Fixed-effects model
Drainage usage	2	0.966	0.653	1.430	0.936	0.816	0.000	Fixed-effects model
Surgeon experience level	2	0.802	0.291	2.208	0.699	0.016	79.008	Random-effects model
Surgery delay > 24 hours	2	0.680	0.461	1.002	0.078	0.721	0.000	Fixed-effects model
Previous surgery at any site	2	0.615	0.381	0.994	0.066	0.856	0.000	Fixed-effects model
Antibiotic prophylaxis usage	2	0.392	0.249	0.618	<0.001	0.177	41.664	Fixed-effects model

LL, lower limit; OR, odds ratio; UL, upper limit; CI, confidence interval; BMI, body mass index; ASA, American Society of Anesthesiologists.

### 3.7 Sensitivity analysis to evaluate the robustness of meta-analysis findings

**Figure 4:**
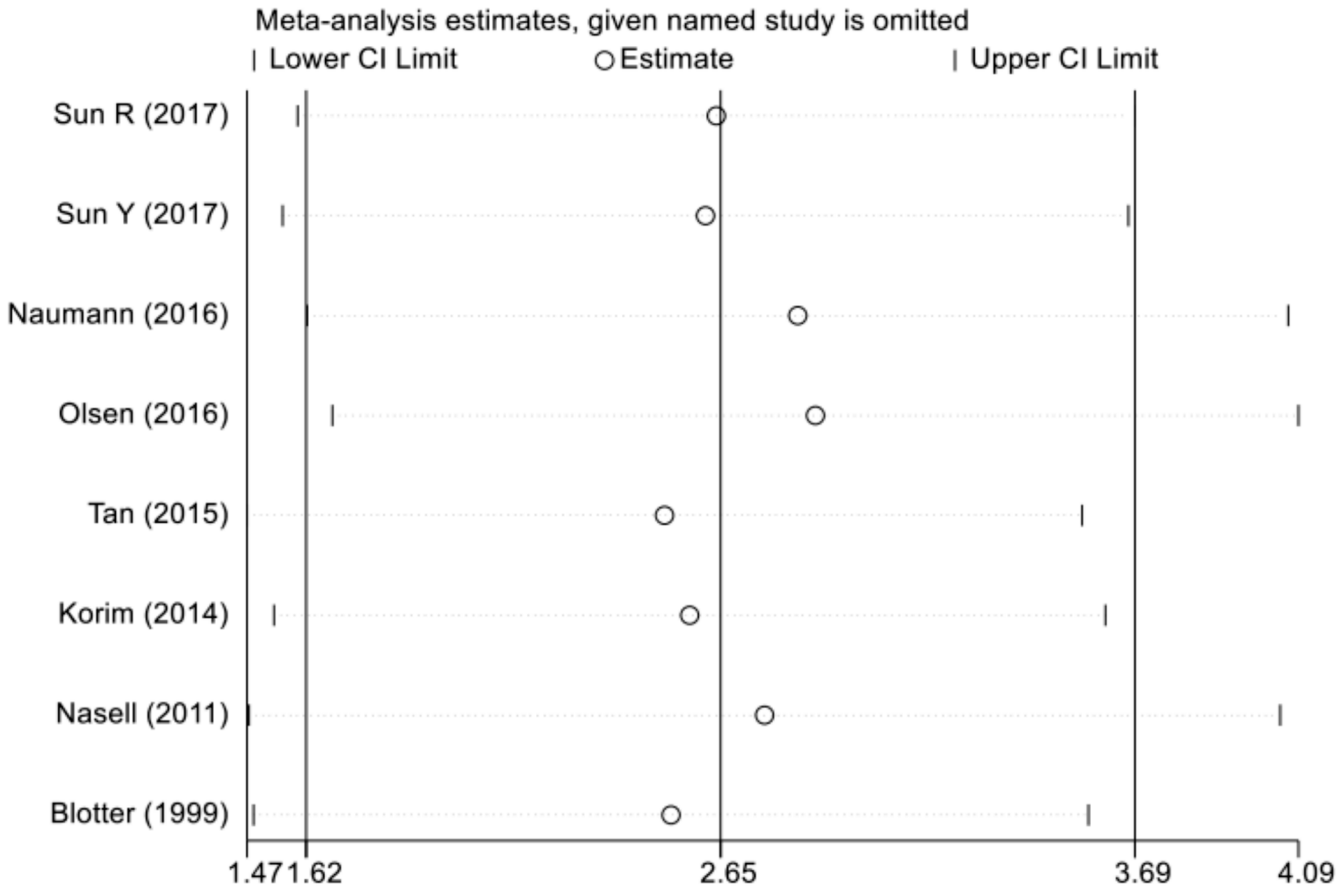
Sensitivity analysis of the association between diabetes and surgical site infections. This plot illustrates the impact of each individual study on the overall pooled odds ratio (OR) through a leave-one-out sensitivity analysis. The open circles (∘) represent the recalculated pooled OR and its 95% confidence interval (CI) when the corresponding study is omitted from the meta-analysis. The vertical solid line indicates the overall pooled OR (2.65) based on all included studies. The x-axis displays the range of recalculated ORs, and tick marks denote key values along this range. Notably, all open circles fall within the 95% CI of the original pooled estimate, indicating that no single study disproportionately influenced the overall result. Wound Infections.

In light of the significant heterogeneity detected across the studies incorporated into our meta-analysis, we undertook a sensitivity analysis to gauge the robustness and consistency of our aggregated findings. This procedure entailed the methodical removal of each study in turn, followed by a re-calculation of the overall effect sizes with the remaining studies. This comprehensive approach to sensitivity analysis affirmed that the aggregate outcomes of our meta-analysis were steadfast and resilient, showing minimal fluctuation in the face of individual study exclusions. The absence of any single study’s disproportionate impact on the collective results bolsters the credibility of our meta-analytic conclusions (Figure 4).

### 3.8 Assessment of publication bias in meta-analysis

Egger’s linear regression test revealed no evidence of significant publication bias across all included meta-analyses (*p* > 0.05 for each comparison), supporting the stability and validity of the pooled estimates.

## 4 Discussion

In the field of orthopedic surgery, surgical site infections continue to pose a significant concern, especially after procedures like ORIF for ankle fractures. In addition to complicating the trajectory of recovery, these infections also place substantial expenses on both patients and healthcare systems. The complex interaction of many risk variables linked to SSIs in the context of ankle fracture surgeries highlights the need for a detailed comprehension of these consequences. Ankle fractures provide distinct challenges due to their intricate nature and the possibility of open injuries, which increase the susceptibility of patients to post-surgical infection risks (18, 19). The meta-analysis provides insights into the complex characteristics of SSIs, with a specific emphasis on the significant influence of diabetes and open fractures. The analysis reveals that individuals with diabetes had a significantly higher chance of developing SSIs, as indicated by an OR of 2.94. The increased susceptibility can be ascribed to various physiological changes associated with diabetes, such as weakened immune function, diminished blood circulation, and impaired wound healing capacity. Moreover, the stress of surgery can exacerbate hyperglycemia and activate systemic inflammatory responses, both of which compromise host defense mechanisms, thereby increasing the risk of postoperative infections at surgical sites.

The perioperative period necessitates strict blood glucose management because to the almost threefold increase in infection risk observed among diabetic patients. Potential strategies for managing diabetes in individuals with poorly managed conditions may involve optimizing glycemic control, using advanced wound care procedures, and maybe increasing the use of prophylactic antibiotics (20, 21). Implementing this customized strategy could reduce the increased risk and enhance surgical results in this susceptible group. The analysis further emphasizes the notable risk linked to open fractures, as indicated by an OR of 3.93. The presence of open fractures inherently exposes the wound to external pollutants, hence significantly elevating the risk of infection. A rigorous strategy to manage open fractures is necessary due to the roughly fourfold increase in risk of SSI in such situations. This approach should include rapid and thorough debridement, timely surgical intervention, and perhaps the utilization of local and systemic antibacterial medicines (22). The utilization of a fixed-effect model in this analysis highlights the constant effect size observed across several research, hence strengthening the strong correlation between open fractures and SSIs.

In addition, the meta-analysis examines a range of risk factors that affect the occurrence of SSI, with the use of external fixators and high-energy injury mechanisms being identified as major determinants. These criteria are likely indicative of the seriousness of the injury and the intricacy of the surgical procedure, both of which might worsen the risk of infection. On the other hand, risk variables that may be changed, such as the cleanliness of incisions, the duration of surgery, and lifestyle factors like smoking and alcohol intake, provide specific targets for initiatives to reduce the risk (23). Enhancing surgical site preparation, minimizing operating duration, and promoting preoperative lifestyle adjustments could together decrease the incidence of SSI. The study reveals that specific criteria, such as the utilization of drainage and the level of experience of surgeons, do not have a substantial influence on the risk landscape. This shows that the association between these variables and the incidence of SSI may be more intricate than previously believed. These findings suggest the need for additional research to explore the relationship between surgical procedures, practitioner expertise, and patient outcomes. Antibiotic prophylaxis is a notable preventive factor that greatly decreases the incidence of SSI (24). This highlights the significant importance of antimicrobial stewardship in the context of surgical treatment, underlining the necessity of implementing evidence-based guidelines to enhance the utilization of antibiotics and mitigate the emergence of resistance.

Based on our findings, the significant risk factors identified—such as diabetes, open fractures, high-energy injury mechanisms, smoking, and BMI > 30—should be integrated into comprehensive perioperative management protocols. Specifically, patients with one or more risk factors can be classified as high risk for SSIs using preoperative scoring systems (e.g., modified surgical risk assessment tools incorporating diabetes status, BMI, fracture classification). This approach enables early identification of patients who may benefit from intensified perioperative care. For patients with diabetes, rigorous glycemic control (target HbA1c < 7%) should be achieved preoperatively, as hyperglycemia is a well-documented modifiable risk factor for impaired wound healing. Given the protective effect of perioperative antibiotics identified in our analysis, clinicians should ensure adherence to evidence-based antibiotic prophylaxis guidelines, including appropriate timing and re-dosing for prolonged procedures. For open fractures, strict adherence to debridement and irrigation protocols, along with early wound coverage strategies, should be emphasized to mitigate infection risk. Collaboration between orthopedic surgeons, infectious disease specialists, endocrinologists, and perioperative nursing teams is recommended to develop individualized care plans for high-risk patients. Incorporating these risk factors into routine clinical decision-making could enhance patient outcomes by enabling targeted interventions to reduce SSI rates in patients undergoing ORIF for ankle fractures.

There are several limitations to this meta-analysis. The relatively small number of included studies restricts statistical power and limits the generalizability of the findings, potentially introducing sampling bias and increasing the risk of type II errors. Most included studies were retrospective in nature, inherently subject to information bias, incomplete data, and uncontrolled confounding, thereby limiting the ability to infer causality. Variability in study designs, patient demographics, surgical techniques, perioperative management, and definitions of surgical site infections (SSIs) introduces clinical and methodological heterogeneity that complicates the interpretation of pooled estimates and may confound observed associations. Although SSI definitions across studies generally aligned with CDC criteria, subtle differences in classification could still lead to misclassification bias. Additionally, the reliance on published data raises concerns regarding potential publication bias, as studies with negative or null findings may be underreported. The limited number of studies further constrains the statistical power of sensitivity analyses and publication bias assessments to detect meaningful trends. Moreover, although most included studies applied multivariate analyses to control for confounding, residual confounding likely remains due to differences in adjustment strategies and inconsistent reporting of covariates. Factors such as injury severity, indication bias, and institutional practices may have influenced the results. To improve future research, well-designed prospective studies with standardized perioperative protocols and robust confounding control methods—such as propensity score matching or IPTW—are recommended. Clear reporting of all adjusted variables will help improve comparability and strengthen the overall evidence on SSI risk following ORIF for ankle fractures.

## 5 Conclusion

Our study elucidates numerous risk factors for surgical site infections post-ankle fracture surgeries, such as diabetes, open fractures, high-energy injuries, smoking, and a BMI exceeding 30, among others. The administration of antibiotic prophylaxis stands out as a protective measure. It is imperative for healthcare providers to acknowledge and address these principal risk factors in the aftermath of ankle fracture surgeries, implementing specific strategies to diminish the incidence of surgical site infections and improve patient care.

## Data Availability

The raw data supporting the conclusions of this article will be made available by the authors, without undue reservation.
