# Supplementary material for: Evaluating risk factors for surgical site infections following open reduction and internal fixation surgery for ankle fractures: a systematic review and meta-analysis
Source: Front Public Health. 2025 Jul 17;13:1558994. doi: 10.3389/fpubh.2025.1558994 (PMC12310692; doi:10.3389/fpubh.2025.1558994)
Supplement: Supplementary file 1 [file Table_1.docx]

Supplementary Table 1 Detailed Search Strategies Used in Each Database.

| Database | Search Strategy |
| --- | --- |
| PubMed | ("Ankle Fractures"[Mesh] OR "ankle fracture*" OR "malleolar fracture*" OR "fracture of the ankle") AND ("Open Reduction and Internal Fixation"[Mesh] OR "ORIF" OR "internal fixation" OR "open reduction") AND ("Surgical Wound Infection"[Mesh] OR "surgical site infection*" OR "postoperative infection*" OR "wound infection*" OR "SSI") AND ("Risk Factors"[Mesh] OR "risk factor*" OR "predictor*" OR "associated factor*" OR "determinant*") |
| Embase | ('ankle fracture'/exp OR 'ankle fracture*' OR 'malleolar fracture*' OR 'fracture of the ankle') AND ('open reduction internal fixation'/exp OR 'open reduction' OR 'internal fixation' OR 'ORIF') AND ('surgical wound infection'/exp OR 'surgical site infection*' OR 'postoperative infection*' OR 'wound infection*' OR 'SSI') AND ('risk factor'/exp OR 'risk factor*' OR 'predictor*' OR 'associated factor*' OR 'determinant*') |
| Web of Science | TS=("ankle fracture*" OR "malleolar fracture*" OR "fracture of the ankle") AND TS=("open reduction" OR "internal fixation" OR "ORIF") AND TS=("surgical site infection*" OR "postoperative infection*" OR "wound infection*" OR "SSI") AND TS=("risk factor*" OR "predictor*" OR "associated factor*" OR "determinant*") |
| Cochrane Library | (MeSH descriptor: [Ankle Fractures] explode all trees) OR ("ankle fracture*" OR "malleolar fracture*" OR "fracture of the ankle") AND (MeSH descriptor: [Open Fracture Reduction, Internal] explode all trees) OR ("open reduction" OR "internal fixation" OR "ORIF") AND (MeSH descriptor: [Surgical Wound Infection] explode all trees) OR ("surgical site infection*" OR "postoperative infection*" OR "wound infection*" OR "SSI") AND (MeSH descriptor: [Risk Factors] explode all trees) OR ("risk factor*" OR "predictor*" OR "associated factor*" OR "determinant*") |
